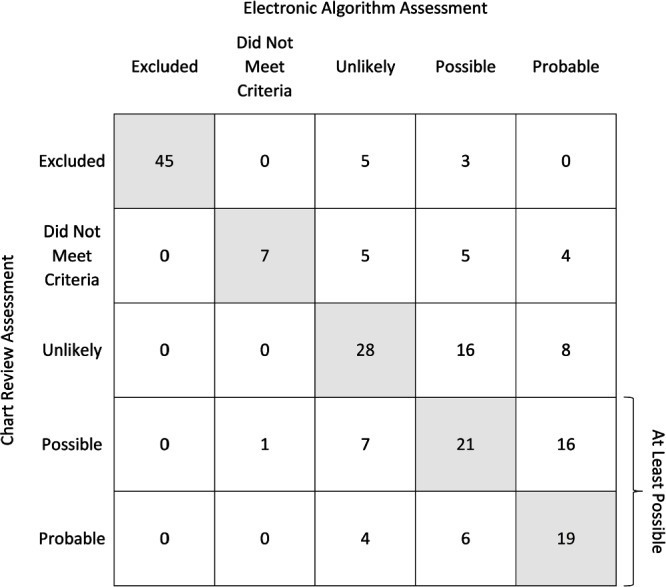# Evaluating the Generalizability of an Electronic Algorithm to Identify Vancomycin-Associated Acute Kidney Injury

**DOI:** 10.1017/ash.2024.170

**Published:** 2024-09-16

**Authors:** Jerald Cherian, Margot Bjoring, Lindsay Donohue, Amy Mathers, Heather Cox, Stacy Park, George Jones, Vorsteg Abigail, Alejandra Salinas, Elizabeth O’Shaughnessy, Ramya Gopinath, Pranita Tamma, Sara Cosgrove, Eili Klein

**Affiliations:** Johns Hopkins University School of Medicine; UVA Health; University of Virginia; University of Virginia Health; The Johns Hopkins University School of Medicine; FDA; Johns Hopkins

## Abstract

**Introduction:** Vancomycin-associated acute kidney injury (V-AKI) is a common adverse reaction; however, there is currently no method to systematically monitor its incidence. We previously developed and internally validated an electronic algorithm to identify cases of V-AKI using structured electronic health record data at the Johns Hopkins Hospital, which demonstrated excellent agreement with chart review (percent agreement 92.5%; weighted kappa coefficient 0.95), as well as excellent sensitivity (89.7%) and specificity (98.2%) in detecting at least possible V-AKI events. The objective of this study was to evaluate the generalizability of the V-AKI electronic algorithm. **Methods:** We identified a retrospective cohort of adult and pediatric patients who received ≥1 dose of intravenous vancomycin while admitted to University of Virginia (UVA) Medical Center from 1/2021-1/2023. An increase in creatinine (Cr) of ≥0.3 mg/dL within 48 hours or ≥50% increase in baseline Cr within 7 days, occurring after the first dose and up to 72 hours after the last dose of IV vancomycin, was considered a potential V-AKI event. The electronic algorithm was executed at UVA with only limited contextualization of hospital specific variables (e.g., procedure names). Patients were categorized as excluded/not meeting criteria, or as having an unlikely, possible or probable V-AKI event using a causality framework. A random subset of the cohort underwent chart review by a blinded reviewer for external validation. Percent agreement and a weighted kappa coefficient were calculated. The sensitivity and specificity in identifying at least possible V-AKI events was determined. **Results:** The electronic algorithm was validated using 200 cases and demonstrated 60.0% percent agreement with chart review (Figure). The weighted kappa coefficient was 0.75. The algorithm was 83.8% sensitive and 71.4% specific in detecting at least possible V-AKI events. Among the 80 discrepant cases, there was only a 1-category difference in 62.5% of cases. The most common reasons for discrepant assessments, which were partly due to inconsistencies in chart review, included disagreement regarding timing of AKI onset (18.6%) and whether renal function returned to baseline (16.3%). **Conclusions:** An electronic algorithm to identify V-AKI events was successfully implemented at another institution. Although agreement with chart review was only fair, sensitivity in detecting at least possible V-AKI events remained excellent. The electronic algorithm may be useful for systematically and reproducibly identifying V-AKI events across institutions in a scalable manner to inform stewardship interventions. However, further refinement of the algorithm and improvement in consistency of chart review assessments is needed.

**Disclosure:** Lindsay Donohue: Advisor – Abbvie

**Figure:**